# HbA1c may contribute to the development of non-alcoholic fatty liver disease even at normal-range levels

**DOI:** 10.1042/BSR20193996

**Published:** 2020-01-31

**Authors:** Changxi Chen, Zhongwei Zhu, Yushan Mao, Yimin Xu, Juan Du, Xiaoping Tang, Hongbao Cao

**Affiliations:** 1 Department of Gastroenterology, Hospital of Zhenhai Refine-Chemical Company, Ningbo, Zhejiang Province 315207, China; 2 Department of Endocrinology, Affiliated Hospital of Medical College of Ningbo University, Ningbo, Zhejiang Province 315020, China; 3 Department of Psychiatry, First Hospital/First Clinical Medical College of Shanxi Medical University, Taiyuan, Shanxi Province 030001, China

**Keywords:** HbA1c, non-alcoholic fatty liver disease, pathway analysis, RAGE

## Abstract

Previous clinical studies highlighted nonalcoholic fatty liver disease (NAFLD) as a hepatic facet of metabolic syndrome, which progresses toward Type 2 diabetes along with an elevation of HbA1c in the blood. Longitudinal observations were performed in a cohort of 2811 participants with no liver disease at inception. The rate of the conversion into NAFLD was 15.7% (440/2811), with a steady increase in prevalence observed in sub-cohorts with increasing HbA1c levels. Moreover, regression analysis indicated that HbA1c levels serve as the risk factors for NAFLD after multiple adjustments (odds ratio: 1.58, *P*-value < 0.004). When HbA1c-related molecular networks were investigated using natural language programming algorithms, multiple genetic/small molecular (SM) pathways were highlighted as connectors between the HbA1c levels and the development of NAFLD, including ones for nitric oxide, hypoxia and receptor for advanced glycation end products (RAGE). Our results suggest that increased levels of HbA1c may contribute to the progression of NAFLD either directly, by stimulating RAGE or indirectly, through the promotion of hypoxia and suppression of the release of NO. Further studies are needed to test the impact of HbA1c on the development of the chronic liver disease.

## Introduction

Nonalcoholic fatty liver disease (NAFLD) is a common chronic disorder characterized by fatty degeneration of hepatocytes, which is not caused by excessive consumption of alcohol. The pathological spectrum of NAFLD starts as simple steatosis, followed by its progression to nonalcoholic steatohepatitis (NASH), which may lead to the development of cirrhosis and/or liver cancer [1]. Being the most common cause of elevated liver enzymes, NAFLD became a major epidemiological concern worldwide [2], with the prevalence in the last decade reaching 12–24% [3,4]. Individuals with NAFLD have a high frequency of metabolic comorbidities to the degree that NAFLD was dubbed a hepatic manifestation of metabolic syndrome [5]. Moreover, NAFLD is also the most common cause of elevated liver enzymes, and thereby became a major epidemiological concern worldwide [2], with the prevalence in the last decade reaching 12–24% [3]. The pathophysiology of NAFLD and its progression to NASH and to liver fibrosis is influenced by both genetics and environmental factors, which augment each other as a set of multiple parallel hits, with oxidative stress as a prominent promoter of the damage to hepatic parenchyma [6,7].

Glycosylated hemoglobin (HbA1c) is a product of the non-enzymatic chemical reaction between glucose and the N-terminus of the h chain (valine) amino groups on hemoglobin protein. From the molecular biology point of view, HbA1c belongs to a diverse group of molecules termed advanced glycation end-products (AGEs). In each individual, diabetic or non-diabetic, relative levels of HbA1c reflect the duration and the severity of hyperglycemic episodes as well as overall exposure to the extracellular glucose present in circulation. At varying cut-offs, the levels of HbA1c were developed as biomarkers both for the diagnosis of diabetes and for the premorbid chronic glycemic control [8,9].

While an association of diabetes with NAFLD and NASH is well documented [10–12], the relationship of the HbA1c levels to the development of NAFLD is less clear. All aspects of the pathophysiology of NAFLD [13], including individuals’ propensity to form advanced glycation end-products (AGE) including HbA1C [14], and the intensity of pro-inflammatory signal produced by binding of AGE to various isoforms of its receptor RAGE [15,16] are influenced by inherited gene variants.

To untangle the complex relationships between HbA1C as a functional molecule and NAFLD, we integrated large-scale literature-based relation data with the clinical and performed a comprehensive multiple level analysis. Obtained results may aid in the understanding of the pathophysiology of both NAFLD and Type 2 diabetes.

## Materials and methods

We studied the association between NAFLD and HbA1c at three levels: first, we conducted a clinical study to dissect the relationship between NAFLD and HbA1c; second, we explored the relationship between NAFDLD and HbA1c as a molecule through a large scale literature-based pathway analysis; finally, we analyzed small molecules connecting elevation of HbA1c levels to the development of NAFLD.

### The clinical study design

#### The participant’s selection

Study participants (*N* = 4905; age range: 45–89) were recruited from the South China area (Ningbo, Zhejiang province) in 2012. The study population included 2753 males and 2152 females. After 3 years of annual follow up (2013, 2014 and 2015) and relative data quality control, 2094 participants were excluded from the study, while 2811 subjects met the criteria of the study (male/female: 1664/1147; sex ratio 1.45: 1; average age: 58.2 ± 9.8 years). The study was reviewed and approved by the Ethics Committee of the Zhenhai Lianhua Hospital, Ningbo, China. Informed consent was obtained from all individual participants. All methods were performed in accordance with the relevant guidelines and regulations.

The exclusion criteria were as follows: (1) participant diagnosed with a specific liver disease, including fatty liver, viral hepatitis, Wilson’s disease, autoimmune liver disease or drug-induced liver disease; (2) participants have a long history of alcohol consumption or consumptions of ethanol were more than 140 g/week for men and more than 70 g/week for women; (3) participants have been recently treated with liver protecting drugs; (4) participants signed the consent but waived the physical examinations; (5) the data collection for a participant was not complete.

#### Diagnosis of NAFLD

The diagnosis of NAFLD was based on the criteria suggested by the Chinese Liver Disease Association (http://www.heporg.com), and the clinical diagnostic standards [17] (Fan et al. 2011) for the determination of NAFLD as described as our previous publication [18] (Chen 2017).

#### Detection of HbA1c

A 10 ml volume of fasting venous blood was drawn from the cubital vein and centrifuged to prepare the serum, which was subsequently used for biochemical analysis and HbA1c index test. The biochemical indices include ALT, AST, GGT, TG, TC, HDL-C, UA, FPG, etc. Each of these biochemical indicators was tested using the same Au640 fully automatic biochemical analyzer (Olympus, Kobe, Japan). HbA1c was tested using an HLC-723 G7 fully automatic HbA1c analyzer (Tosoh Chemical Company, Tokyo, Japan), and reagents and quality control products were provided by the same company. The analysis was conducted by experimental methodological tests.

### Statistical analysis

Statistical analysis was performed by the SPSS 18.0 software package (IBM Corp., Armonk, NY, U.S.A.). The variables were expressed as the mean ± standard deviation or median ± quartile interval. Continuous variables were compared using the *T*-test (normal distribution) or Mann–Whitney test (not consistent with normal distribution). The chi-square test was used to examine categorical data rates. The Cox proportional hazards model was used to evaluate the risk factors for NAFLD, with the following factors included prior to running the analysis: age, gender, waist circumference, body mass index, systolic pressure, diastolic pressure, total cholesterol, triacylglycerol, low-density lipoprotein cholesterol, fasting blood glucose, high-density lipoprotein cholesterol and serum HbA1c. The difference was considered to be statistically significant when *P* < 0.05.

### HbA1c–gene and NAFLD–gene relation data acquisition

We acquired the HbA1c–gene and NAFLD–gene relation data from Pathway Studio (www.pathwaystudio.com) and presented the related information in a searchable table file HbA1c_NAFLD, which is publicly available at (http://gousinfo.com/database/Data_Genetic/HbA1c_NAFLD.xlsx). The set of genes with shared association with both NAFLD and HbA1c was analyzed with Gene Set Enrichment Analysis (GSEA) against Gene Ontology (GO) and Pathway Studio Ontology (PSO).

### The study of the compounds acting upon HbA1c and NAFLD

The relationship of HbA1c and NAFLD was explored by querying a set of compounds with evidence of influencing either levels of HbA1C or the NAFLD or both. The relation data were also acquired from Pathway Studio as described above, with all supporting data provided in the database HbA1c_NAFLD.

### Shortest-path analysis

Based on the molecular relations identified, pathways that connecting HbA1c and NAFLD were build using the ‘Shortest-Path’ model within the Pathway Studio environment (www.pathwaystudio.com), with the purpose to identify genes and compounds/drugs, through which HbA1c and NAFLD may influence each other. The information of the references supporting the relationships in a pathway was provided in the Supplementary Material (HbA1c_NAFLD). Within Pathway Studio, there is a Confidence Score to evaluate the quality of a relationship. The higher the score, the higher the quality of a relation.

## Result

### Patients with ‘upper normal’ HbA1C levels are more likely to develop NAFLD than patients with ‘core normal’ levels

This prospective study aimed at detecting the accumulation of NAFLD diagnosis in a cohort of patients with healthy livers and normal levels of HbA1c (5.6% or less) levels of HbA1c at baseline. Notably, among the 2811 participants of the present study, we have identified 193 subjects with healthy liver and abnormal (pre-diabetic) levels of HbA1c (between 5.7% and 6.5%). On average, patients with abnormal levels of HbA1c at baseline were older (*N* = 193; mean age: 64.3 ± 10.4 years) than those with the levels of HbA1c falling within a normal range (*N* = 2618, mean age: 57.8 ± 10.2 years). At the end of 3 years follow-up, the cumulative incidence of NAFLD was 440/2811 cases (15.7%), including 285 males (17.1%) and 155 females (13.5%). As expected, the incidence of NAFLD in patients with normal levels of HbA1c was lower (391/2618 or 14.9%) than that in the pre-diabetic group (49/193 or 25.4%) (χ^2^ = 6.717, *P* < 0.010).

At the next stage of analysis, patients with normal levels of HbA1c were further subdivided into ‘upper normal’ (HbA1c between 5.4% and 5.6%, *N* = 773) and ‘core normal’ (HbA1c of 5.3% or less, *N* = 1411) groups. As could be seen from Table 1, the levels of HbA1c continued to have a significant relation with the incidence rate of NAFLD when all three groups were compared (d*f* = 2, *χ*^2^ = 8.59, *P* < 0.014).

**Table 1:** The incidence of NAFLD with different levels of HbA1c

	Percentage of glycosylated hemoglobin			
	≤5.3%	>5.3% - ≤5.6%	5.7–6.5%	Total
Did not develop NAFLD	1213	651	507	2371
Developed NAFLD	198	122	120	440
Incidence rate	14.03%	15.78%	19.14%	15.65%

### The relationship between the levels of HbA1c and the likelihood to develop NAFLD is linear

To eliminate confounding effects of other risk factors when considering the influence of the levels of HbA1c on NAFLD, the relationships between NAFLD and each clinical parameter were tested in the multivariate Cox proportional hazards regression model. As could be seen in Table 2, maximally adjusted model 4 suggests that NAFLD is more likely to occur in individuals with elevated HbA1c levels, with the odds ratio of 1.58, and *P*-value < 0.004. Other parameters contributing to the risk of NAFLD included gender, age, the levels of uric acid, AST, ALT and triglycerides as well as body mass index and waist circumference (all *P*-values < 0.05) (Table 2).

**Table 2:** Multivariate Cox proportional hazards regression analyses in entire cohort

	β	SE	Wald x^2^ value	*P* value	OR	95% CI
Model 1	0.586	0.152	14.935	<0.001	1.796	1.335–2.418
Model 2	0.674	0.155	18.956	<0.001	1.963	1.449–2.659
Model 3	0.587	0.157	14.07	<0.001	1.799	1.324–2.445
Model 4	0.458	0.188	8.454	<0.005	1.580	1.161–2.152

Note: Model 1 was unadjusted. Model 2 was adjusted for age and sex. Model 3 was adjusted for age, sex and body mass index. Model 4 was adjusted for all Model 3 variables associated plus these associated with metabolic syndrome, including waist circumference, systolic blood pressure, diastolic blood pressure, triglycerides, HDL cholesterol, and fasting blood sugar. β is partial regression coefficient; SE is standard error of partial regression coefficient; OR is odds ratio; CI is confidence interval. *P*-value specifies the possibility that a parameter is not a risk factor for NAFLD.

#### Other potential influential factors of NAFLD

During the 3-year follow-up period, 440 incident NAFLD cases (285 men and 155 women) were identified, and the cumulative incidence was 15.7% (440/2811). The baseline characteristics of participants were compared according to follow-up outcomes. Compared with remained NAFLD-free participants, incident NAFLD participants had higher baseline waist circumference, body mass index, systolic and diastolic blood pressure, triacylglycerol, total and low-density lipoprotein cholesterol, glutamyl-transpeptidase, fasting blood glucose, uric acid, hemoglobin and HbA1c levels, while lower baseline high-density lipoprotein cholesterol levels (Table 3).

**Table 3:** Comparison of baseline characteristics of participates according to follow-up outcomes

Variable	Did not develop NAFLD (*n* = 2371)	Developed NAFLD (*n* = 440)	*t* value	*P* value
Age (year)	58.4±10.0	57.2±9.2	−2.42	0.016
Waist circumference (cm)	80.6±8.2	85.8±7.6	12.2+	<0.001
Body mass index (kg/m^2^)	22.5±2.5	24.5±2.4	15.21	<0.001
Systolic blood pressure (mmHg)	125.6±16.2	128.9±16.3	3.87	<0.001
Diastolic blood pressure (mmHg)	77.8±9.5	80.0±9.7	4.39	<0.001
Total cholesterol (mmol/l)	5.1 (4.5,5.7)	5.3 (4.6,5.9)	3.05*	0.002
Triglycerides (mmol/l)	1.0 (0.7,1.3)	1.3 (1.0,1.8)	12.34*	<0.001
HDL cholesterol (mmol/l)	1.7±0.4	1.5±0.3	−9.73	<0.001
LDL cholesterol (mmol/l)	2.9±0.7	3.1±0.8	3.87	<0.001
Glutamine transpeptidase (U/l)	19 (14,26)	25 (18,34)	9.83*	<0.001
Fasting blood sugar (mmol/l)	5.2±0.9	5.4±1.1	2.56	0.010
Uric acid (μmol/l)	287.6±75.5	315.4±74.5	7.11	<0.001
Hemoglobin (g/l)	133.6±13.7	137.4±12.9	5.41	<0.001
Glycosylated hemoglobin (%)	5.4±0.5	5.5±0.7	2.59	0.010

Abbreviations: *, *z* value; HDL, high-density lipoprotein; LDL, low-density lipoprotein

### Genes contributing to the levels of HbA1c and to NAFLD

Pathway Studio-guided analysis highlighted a total of 71 genes associated with the levels of HbA1c. This list of genes was supported by 141 peer-reviewed sources published from 1982 to 2018. For NAFLD, we identified 898 associated genes, supported by over 4000 references published from 1999 to 2018. A significant overlap of 54 genes (Fisher-Exact test *P*-value<1.82e-65) between the HbA1c- and NAFLD-associated gene sets was detected. This overlap covered approximately 76.1% of the HbA1c gene set (54/71) and 6.01% of the NAFLD gene set (54/898). For the list of these 54 genes and the supporting reference, please refer to HbA1c_NAFLD→ 54 Common Genes and HbA1c_NAFLD→ Ref for 54 Common Genes.

A Gene Set Enrichment Analysis (GSEA) was conducted to evaluate the functional profile of the 54 genes associated with both HbA1c and NAFLD. The top 10 significantly enriched pathways (FDR-corrected p-value≤7e-15) are presented in Table 4. The complete list of 29 pathways/gene sets with p-values for their enrichment below the cut-off of < 10e-10 are provided in HbA1c_NAFLD→ GSEA.

**Table 4:** Functional pathways enriched by 54 genes contributing to both NAFLD and HbA1c levels

Name	Overlap genes	Jaccard similarity	P-value after FDR	P-value before FDR
Response to nutrient levels	24	0.040268	2.07E-20	1.55E-24
Response to extracellular stimulus	24	0.038339	5.3E-20	5.28E-24
Response to hormone	26	0.0282	9.73E-19	1.46E-22
Insulin -> STAT Expression Targets	20	0.103627	1.96E-17	6.84E-21
Response to peptide hormone	19	0.04034	3.68E-16	1.84E-19
Insulin -> CEBPA/CTNNB/FOXA/FOXO Expression Targets	19	0.093137	1.43E-15	8.19E-19
Response to peptide	19	0.036538	2.25E-15	1.35E-18
Aging	18	0.040359	2.78E-15	1.74E-18
Insulin -> MEF/MYOD Expression Targets	19	0.090047	2.78E-15	1.8E-18
Insulin -> ELK/SRF/HIF1A/MYC/SREBF Expression Targets	19	0.086364	6.77E-15	4.73E-18

### Possible co-regulatory events shared for HbA1c levels and NAFLD

**Figure 1:**
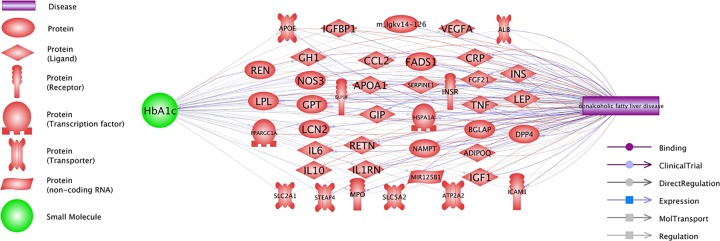
Positive co-regulation of the levels of HbA1c and the development of NAFLD

Pathway Studio guided Shortest-Path analysis identified multiple molecular networks, through which the levels of HbA1c and the development of NAFLD may be mutually influencing each other (Figure 1). These networks encompass 41 out of 54 shared contributors, with a total of 97 relations supported by 1001 references (Figure 1). For the detailed description of each of these HbA1c–gene–NAFLD relations, please refer to HbA1c_NAFLD→Co-regulation Network, where the supporting references and corresponding sentences are systematically described**.**

### Small molecules capable of influencing both the development of NAFLD and the levels of HbA1C

**Figure 2:**
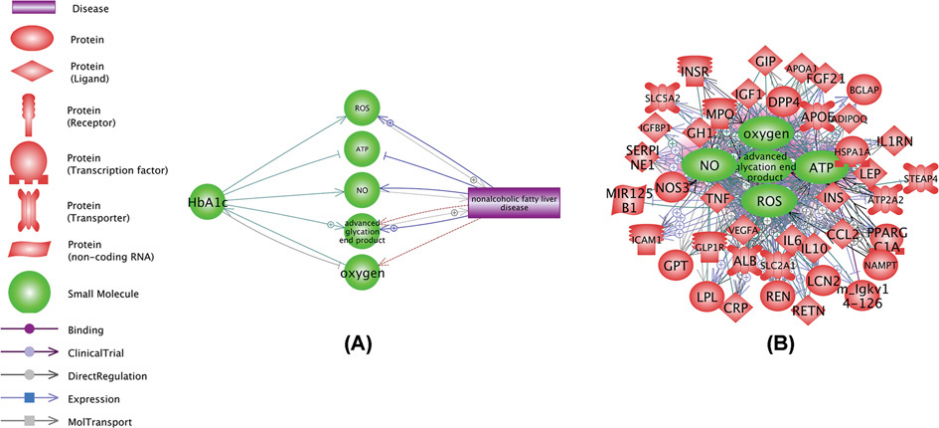
Small molecules contributing to, or responding to both the levels of HbA1c and the development of NAFLD (**A**) The levels of HbA1c and the development of NAFLD are connected by 5 molecules. (**B**) Relation network which integrated 5 small molecules highlighted by the ‘Shortest Path’ analysis, and 40 out of 41 genes that connect elevated levels of HbA1c and the development of NAFLD.

Figure 2A shows an output of ‘Shortest Path’ analysis, which identified 5 small molecules/ drugs bridging HbA1c and NAFLD. The relations presented in Figure 2A were supported by 237 references (see HbA1c_NAFLD→Ref for 5 SMs).

Notably, 40 out of 41 genes in Figure 1 also displayed a strong set of relations with all the 5 small molecular interactors (Figure 2B). These relations were supported by over 10,000 references. For the detailed description of the disease–SM–gene relations, please refer HbA1c_NAFLD→5 SMs_41 Genes.

## Discussion

Blood levels of glycated hemoglobin (HbA1c) are commonly used as a screening tool for detecting insulin resistance in general, and the presence of Type 2 diabetes [19]. Insulin resistance is a known pathophysiological driver of NAFLD [20–22]. Recent studies suggest that the levels of HbA1c levels may contribute to the development of NAFLD directly [10,11]. In this work, and also some previous works, an association of HbA1c levels and the risk for NAFLD was demonstrated for nondiabetic individuals, independently of obesity and other metabolic components [11]. Notably, the same study also showed that the magnitude of association of HOMA-IR scores with HbA1c levels in subjects already diagnosed with NAFLD is greater than that in those with healthy livers [11].

We confirmed the association between the levels of HbA1c and NAFLD in metabolically intact patients (HbA1C levels of 5.6% or lower). The rate of the conversion into NAFLD was 15.7% (440/2811) for an entire cohort, with a steady increase in prevalence observed in sub-cohorts with increasing HbA1c levels. Moreover, regression analysis highlighted HbA1c served the risk factors for NAFLD after multiple adjustments (odds ratio: 1.58, *P*-value < 0.004).

We have also explored the relationship between the levels of HbA1c and the development of NAFLD by systematic mining of existing literature, and have identified multiple pathophysiologically relevant connections. In particular, disease–gene relation data analysis showed that a majority of the genes associated with the levels of HbA1c were also implicated with NAFLD, with a significant overlap between two gene sets (*P*-value < 1.82e-65). Genes that belong to this overlap were significantly enriched in pathways critical for the response to nutrient levels [23,24] and hormonal stimulation [25,26] (see Table 4 and HbA1c_NAFLD→GSEA).

Further analysis demonstrated that the levels of HbA1c and the development of NAFLD are mutually and positively influencing each other. For example, one of the pathways presented in Figure 2 is HbA1c→NOS3→NAFLD. It has been shown that increased concentrations of HbA1c are capable of significantly down-regulating the expression of NOS3 [27]; resultant deficiency of NOS3 is a known contributor to NAFLD [28]. Another example is the HbA1c→ IL10→NAFLD pathway. While IL10 plays a protective role in NAFLD pathogenesis [29], high concentrations of HbA1c influence the production of this molecule negatively and precipitate the development of the disease [30]. The discovery of these connecting pathways suggests an engagement of a positive feedback loop. For the information on other connecting pathways presented in Figure 2, please refer to HbA1c_NAFLD→ Co-regulation Network.

A set of 5 small molecules related to both the levels of HbA1c and NAFLD (Figure 2A) and related literature are summarized in HbA1c_NAFLD→Ref for 5 SMs. Elevated amounts of HbA1c lead to a decrease in the production of nitric oxide (NO) [31] and the development of the state of hypoxia [32], which both have important implications for NAFLD [33,34]. Further, a recent study showed that persistent elevation of HbA1c levels could lead to the production and accumulation of advanced glycation end products (AGE) [35], yet another NAFLD contributor [36]. As HbA1c is one of high volume AGE products, Hb-AGE, it is worthwhile to note that a close correlation of the amounts of HbA1c and Hb-AGE across diabetic subjects was previously observed, with the magnitude of this effect increases in the cohorts with least degree of glycemic control [37].

Additionally, we noted that 40 out of 41 genes included in the HbA1c→gene→NAFLD pathways (Figure 1) also had demonstrated strong associations with all the 5 drugs presented at Figure 2A, with a total of 317 relations supported by over 10,000 references (see Figure 2B and HbA1c_NAFLD→5 SMs_41 Genes). The remarkable density of gene–small molecule interaction networks that connects both HbA1a and NAFLD indicates close, possibly functional inter-relationship between these two aspects of metabolic syndrome. Biological mechanisms mediating HbA1c→gene/SM→NAFLD network are worthy of further study.

One limitation of the present study was that the clinical data were short of insulin resistance (IR) measurement, which could be a causal factor that leads to increased HbA1c levels and NAFLD incidence. Therefore, we speculate that the HbA1c level in the range of 5.4 to 5.6% may be reflective of early insulin resistance and thus may not be entirely healthy. Further study is needed to test the impact of IR on the HbA1c−NAFLD association observed here.

## Conclusion

The results of the present study suggested that increased levels of HbA1c may contribute to the development of NAFLD. This form of altered hemoglobin may contribute to the progression of NAFLD either directly, by stimulating RAGE or indirectly, through the promotion of hypoxia and suppression of the release of NO. Multiple molecular pathways identified by applying natural language programming algorithms may aid in improving our understanding of the underlying pathophysiology of NAFLD.

## Data Availability

Data are available by contacting the corresponding author.

## Abbreviations

NAFLD: nonalcoholic fatty liver disease
RAGE: receptor for advanced glycation end products
SM: small molecular
